# Decreased expression of *femXAB* genes and *fnbp* mediated biofilm pathways in OS-MRSA clinical isolates

**DOI:** 10.1038/s41598-019-52557-z

**Published:** 2019-11-05

**Authors:** Umarani Brahma, Paresh Sharma, Shweta Murthy, Savitri Sharma, Shalini Chakraborty, Sundarapu Naga Appalaraju, Vasundhra Bhandari

**Affiliations:** 1 National Institute of Animal Biotechnology, Hyderabad, India; 2 L.V. Prasad Eye Institute, Hyderabad, India

**Keywords:** Antimicrobial resistance, Clinical microbiology

## Abstract

Methicillin-Resistant *Staphylococcus aureus* (MRSA) is a significant threat to human health. Additionally, biofilm forming bacteria becomes more tolerant to antibiotics and act as bacterial reservoir leading to chronic infection. In this study, we characterised the antibiotic susceptibility, biofilm production and sequence types (ST) of 74 randomly selected clinical isolates of *S. aureus* causing ocular infections. Antibiotic susceptibility revealed 74% of the isolates as resistant against one or two antibiotics, followed by 16% multidrug-resistant isolates (MDR), and 10% sensitive. The isolates were characterized as MRSA (n = 15), Methicillin-sensitive *S. aureus* (MSSA, n = 48) and oxacillin susceptible *mecA* positive *S. aureus* (OS-MRSA, n = 11) based on oxacillin susceptibility, *mecA* gene PCR and PBP2a agglutination test. All OS-MRSA would have been misclassified as MSSA on the basis of susceptibility test. Therefore, both phenotypic and genotypic tests should be included to prevent strain misrepresentation. In addition, in-depth studies for understanding the emerging OS-MRSA phenotype is required. The role of *fem XAB* gene family has been earlier reported in OS-MRSA phenotype. Sequence analysis of the *fem XAB* genes revealed mutations in *fem* × (K3R, H11N, N18H and I51V) and *fem B* (L410F) genes. The *fem XAB* genes were also found down-regulated in OS-MRSA isolates in comparison to MRSA. In OS-MRSA isolates, biofilm formation is regulated by fibronectin binding proteins A & B. Molecular typing of the isolates revealed genetic diversity. All the isolates produced biofilm, however, MRSA isolates with strong biofilm phenotype represent a worrisome situation and may even result in treatment failure.

## Introduction

*Staphylococcus aureus* causes a wide range of diseases in both humans and animals^1^. It is an opportunistic pathogen, known to acquire resistance against several classes of antibiotics. Methicillin-Resistant *S. aureus* (MRSA) is defined as strains with an oxacillin minimum inhibitory concentration (MIC) of ≥4 mg/L^2^. Most of the MRSA strains contain the *mecA* gene located on the staphylococcal cassette chromosome *mec* (SCC*mec*) which encodes for a modified penicillin-binding protein 2a (PBP2a)^2^. MRSA has emerged as a huge threat around the globe as community, hospital, and livestock acquired infections^2,3^. The surveillance network in the United States claims the MRSA increase from 29.5% in 2000 to 41.6% in 2005^4^. Whereas a report in 2013 from India showed an increase in MRSA prevalence among non-ICU inpatients and ICU patients to be 42 and 43%, respectively in 2008 as compared to 49 and 47%, in 2009, respectively^5^. There is also an increasing prevalence of MRSA in ocular infections which is alarming^4,6,7^. Reports suggest MRSA infections affect the neonates and aged individuals at a more aggressive rate because of their comparatively decreased immunity^8,9^ common surgeries such as photo reactive or refractive keratectomies often are paving the way for MRSA infectious keratitis making it an imperceptibly tricky situation for the patients and doctors alike^10,11^. Another recent unnerving revelation is that *S. aureus* may disseminate from the blood to eye in patients suffering from bacteraemia^12^.

Recently, OS-MRSA (Oxacillin susceptible *mecA* positive *S. aureus*) clinical isolates have surfaced across the globe which possess the *mecA* gene but are susceptible to oxacillin^2,3^. However, not much data is yet available on the prevalence, virulence, resistance mechanism of OS-MRSA strains and their impact on the current treatment regimen. Another imperative should be the molecular characterisation of existing clinical isolates.

In addition to the intriguing phenotype of *S. aureus*, their capacity to form biofilms on biotic or abiotic surfaces especially, medical devices, catheter, ocular implants and corneal stroma further magnify the problem^13^. Biofilm formation is an innate component of the prokaryotic life cycle which ensures the survival of the bacterial cells by fending off host immune attacks as well as external therapeutic/antibiotics and also promote dispersal to other uninfected parts thereby maintaining a chronic infection^14^. We hence need to identify and understand the molecular characteristics of such bacterial populations to unarm them. In the current study, we are trying to understand the molecular characteristic, resistance and biofilm profiles of MRSA and MSSA strains isolated from ocular infections and to determine the prevalence of OS-MRSA which is not well understood.

## Materials and Methods

### Isolation of *S. aureus* from clinical samples

Clinical isolates of randomly selected *S. aureus* were received from L.V Prasad Eye Institute (LVPEI), Hyderabad, India. Further, species identification and characterisation of *S. aureus* cultures were performed using various biochemical and molecular tests as discussed earlier^3^. Following an overnight incubation in trypticase soy broth (TSB) at 37 °C, the cultures were streaked on Baird Parker agar plates (30% egg yolk emulsion and 1% potassium tellurite) and after that onto mannitol salt agar plates. Yellow colonies on the plates were considered as *S. aureus* and inoculated further in TSB for other biochemical tests such as catalase, Latex agglutination, Coagulase test and Gram staining. Species confirmation was also done by sequence analysis of the amplified PCR product of *16S rRNA* gene as mentioned below.

### PCR amplification

Genomic DNA was isolated using the Wizard genomic kit (Promega) as per manufacturer’s instructions. Briefly, the overnight grown culture in TSB media was centrifuged at 3200 × g for 10 minutes and washed twice in PBS. Cells were pelleted and resuspended in 500 µl lysis buffer (50 mM EDTA) and 50 µl of lysozyme (20 mg/ml) and incubated at 37 °C for 2 hrs. PCR was performed for *16S rRNA*, *mecA, mecC and SCCmec* types and *agr* typing as described previously^3,15–18^.

### Multilocus sequence typing (MLST)

Internal fragments of seven housekeeping genes, Carbamate kinase (*arc*), Shikimate dehydrogenase (*aroE*), Glycerol kinase (*glpF*), Guanylate kinase (*gmk*), Phosphate acetyltransferase (*pta*), Triosephosphate isomerase (*tpi*), and Acetyl coenzyme A acetyltransferase (*yqiL*) were amplified using the approach described on http://www.mlst.net/. The sequence types (STs) were designated by comparing the data of seven sequenced internal fragments of each strain with the *S. aureus* MLST database (http://www.mlst.net/).

Global optimal eBURST (goeBURST) of identified STs, was analysed using the PHYLOViZ online software^19^. *S. aureus* MLST public database was used to make the minimum spanning tree based on the goeBURST analysis for the identified STs. The nLocus Variant (nLV) was kept to 2 to keep all STs with a distance equal to or below 2 connected in the tree.

### Antibiotic susceptibility profile

The antibiotic susceptibility profile was determined using the disk diffusion method and microbroth dilution assay as per CLSI guidelines with minor modifications as mentioned before^18^. Briefly, the overnight grown culture was adjusted to 0.5 McFarland standard (Himedia, Mumbai, India) and was streaked onto Mueller–Hinton Agar (Himedia, Mumbai, India) plates for disk diffusion while Mueller-Hinton broth was used for micro-broth dilution assay. The disk diffusion assay was done against tetracycline (30 µg), gentamicin (10 µg), rifampicin (5 µg), erythromycin (15 µg), ciprofloxacin (10 µg), clindamycin (2 µg), and cefoxitin (30 µg). Resazurin dye based microbroth dilution assay was performed against oxacillin, vancomycin and linezolid^18^. ATCC 29213 (methicillin sensitive control strain) and ATCC 700699 (methicillin-resistant control strain with reduced vancomycin susceptibility) were used as a control for these assays^3,18^. Clinical isolates showing resistance against three or more class of antibiotics were described as multidrug-resistant (MDR), resistance against one or two class of antibiotic was referred as resistant and sensitivity towards all antibiotics was mentioned as pan-sensitive in the result section.

### PBP2a Latex Agglutination Test

PBP2a, which is the *mecA* gene product was detected using Oxoid PBP2′ Latex Agglutination Test’ kit (DR0900A, Thermo Scientific™), following the manufacturer’s instructions. ATCC 43300 and ATCC 29213 were used as controls.

### Sequence analysis of *fem XAB* family

The *fem XAB* gene family was amplified as described earlier^20^. The primers used for amplification of *fem* genes were as follows: *femX* (For-GCCATGGAAAAGATGCATATCAC and Rev-CTCGAGTTTTCGTTTTAATTTACGAG), *femA* (For-GGATCCATGAAGTTTACAAATTTAACAGC Rev-CTCGAGAAAAATTCTGTCTTTAAGTTTTTTAAG) and *femB* (GGATCCATGAAGTTTACAAATTTAACAGC and Rev-CTCGAGTTTCTTTAATTTTTTACGTAATTTATCG)^20^. The amplified PCR product was sequenced using forward and reverse primer along with the internal primers for all the respective genes (Internal *femA*-GATCCGTGCTACAAATTCGT; internal *femX*-CACCAATTGATAAAAATGATG and internal *femB*-CAACTGAG TATGATACATCGA). The data was analysed for mutation using DNA star software (SeqMan NGen®. Version 12.0. DNASTAR. Madison, WI.).

### Expression analysis of *fem XAB* and biofilm-related genes

RNA was isolated using the Macherey Nagel RNA isolation kit as per the manufacturer’s instructions (NucleoSpin^®^ RNA, 740955.10). Approximately from 2.5 µg of RNA, cDNA was synthesised using the Clontech cDNA synthesis kit (PrimeScript™ 1st strand cDNA Synthesis Kit, 6110B). SYBR green-based relative gene expression analysis was carried out using the comparative 2^−∆∆^C_t_ method using the ABI real-time detection system (7500, ABI). Real-time PCR was performed for *fem XAB* genes and biofilm-related genes (*icaADBC* and *fnbp A* & *B*). The *gyrB* gene was used as internal control in the study. All the primers used in the study are mentioned in Table 1. All the experiments were repeated thrice.

**Table 1:** List of real-time PCR primers used in the study.

S. No	Primer	Forward sequence (5′-3′)	Reverse sequence (5′-3′)
1.	*gyrB*	CCAGGTAAATTAGCCGATTGC	AAATCGCCTGCGTTCTAGAG
2.	*icaA*	GAGGTAAAGCCAACGCACTC	CCTGTAACCGCACCAAGTTT
3.	*icaB*	ATACCGGCGACTGGGTTTAT	TTGCAAATCGTGGGTATGTGT
4.	*icaC*	CTTGGGTATTTGCACGCATT	GCAATATCATGCCGACACCT
5.	*icaD*	ACCCAACGCTAAAATCATCG	GCGAAAATGCCCATAGTTTC
6.	*fnbp A*	AAATTGGGAGCAGCATCAGT	GCAGCTGAATTCCCATTTTC
7.	*fnbp B*	GCAGCTGAATTCCCATTTTC	ACCTTCTGCATGACCTTCTGCACCT
8.	*femA*	TCGATCCATATTTACCATATCAATACTTG	TCCTAAGTTACTCATTTTATCAAAGAAC
9.	*femB*	TCGTGCCATTTGAAGGTCG	TCAAGGTTTAATACGCCCATCC
10.	*femX*	GCGAAGAATCGCTGTAGGTC	TGCATACGCTTTCTCAGCTT

### Biofilm profiling by crystal violet assay

The biofilm forming capacity of each strain was determined using crystal violet (CV) assay as described previously, ATCC 29213 and ATCC 33592 were used as controls^21^. Briefly, the overnight culture was diluted in TSB (1:100), and dispensed in 96 well plate (200 µl/well). The wells with only broth were treated as the control. After incubation for 18–24 hrs at 37**°**C, the plate was washed 3 times with PBS to remove planktonic cells or media particles. Methanol was added to each well for 15 minutes and then air-dried for 30 minutes. 0.1% crystal violet solution was added to each well and incubated at room temperature for 20 minutes followed by washing using distilled water. After washing, 33% acetic acid was added to each well and absorbance was taken at 590 nm. Mean absorbance values of each sample was calculated and compared with the mean values of controls. All experiments were repeated thrice in quadruplicates. The data were analyzed as described before^22^.

The following conditions were used to interpret the results:

$$\begin{array}{l}{{\rm{O.Dc}} \ge {\rm{O.Ds}} = > {\rm{non}-{adherent}}.}\\{{\rm{O.Dc}} < {\rm{O.Ds}}\le 2\ast {\rm{O.Dc}}= > {\rm{weakly}}\,{\rm{adherent}}.}\\{2\ast{\rm{O.Dc}} < {\rm{O.Ds}}\le 4\ast {\rm{O.Dc}}= > {\rm{moderately}}\,{\rm{adherent}}.}\\{4\ast{\rm{O.Dc}} < {\rm{O.Ds}}= > {\rm{strongly}}\,{\rm{adherent}}.}\\{[{\rm{O.Dc}}= > {\rm{Optical}}\,{\rm{Density}}\,{\rm{of}}\,{\rm{control}};\,{\rm{O.Ds}}= > {\rm{Optical}}\,{\rm{Density}}\,{\rm{of}}\,{\rm{sample}}]}\\\end{array}$$

### Congo-Red assay

Congo red plates were prepared using Brain Heart infusion agar (52 gm/L), sucrose (50 gm/L), congo-red stain (0.8 gm/ml). Overnight cultures of the samples in TSB was inoculated in MHB and kept in the incubator-shaker till they reached an O.D_600nm_ of 0.08–0.13. The cultures were streaked on congo-red agar plates and incubated for 16–18 hours. Plates with black colonies were considered as Congo red positive.

## Results

### Antibiotic susceptibility profile

The antibiotic susceptibility assays were performed for 74 clinical isolates of *S. aureus* against 10 antibiotics (Table 2). High resistance rates were observed against ciprofloxacin (85%) and erythromycin (50%). Moderate resistance was observed against gentamicin (23%) and oxacillin/cefoxitin (20%). However, low resistance was observed against tetracycline (12%), clindamycin (9%) and rifampicin (5%). All the clinical isolates were susceptible to vancomycin and linezolid. Overall, 16% of the isolates were multidrug resistant followed by 74% resistant, and 10% pan-sensitive isolates.

**Table 2:** Overall susceptibility profile of the clinical isolates against tested antibiotics.

Antibiotic	Number of resistant isolates (%)	Number of sensitive isolates (%)
Oxacillin/Cefoxitin	15(20)	59(80)
Linezolid	0(0)	74(100)
Gentamycin	17(23)	57(77)
Erythromycin	37(50)	37(50)
Rifampicin	4(5)	70(95)
Tetracycline	9(12)	65(88)
Clindamycin	7(9)	67(91)
Ciprofloxacin	63(85)	11(15)
Vancomycin	0 (0)	74(100)

### Different STs of *S. aureus* persists in ocular infections

**Figure 1:**
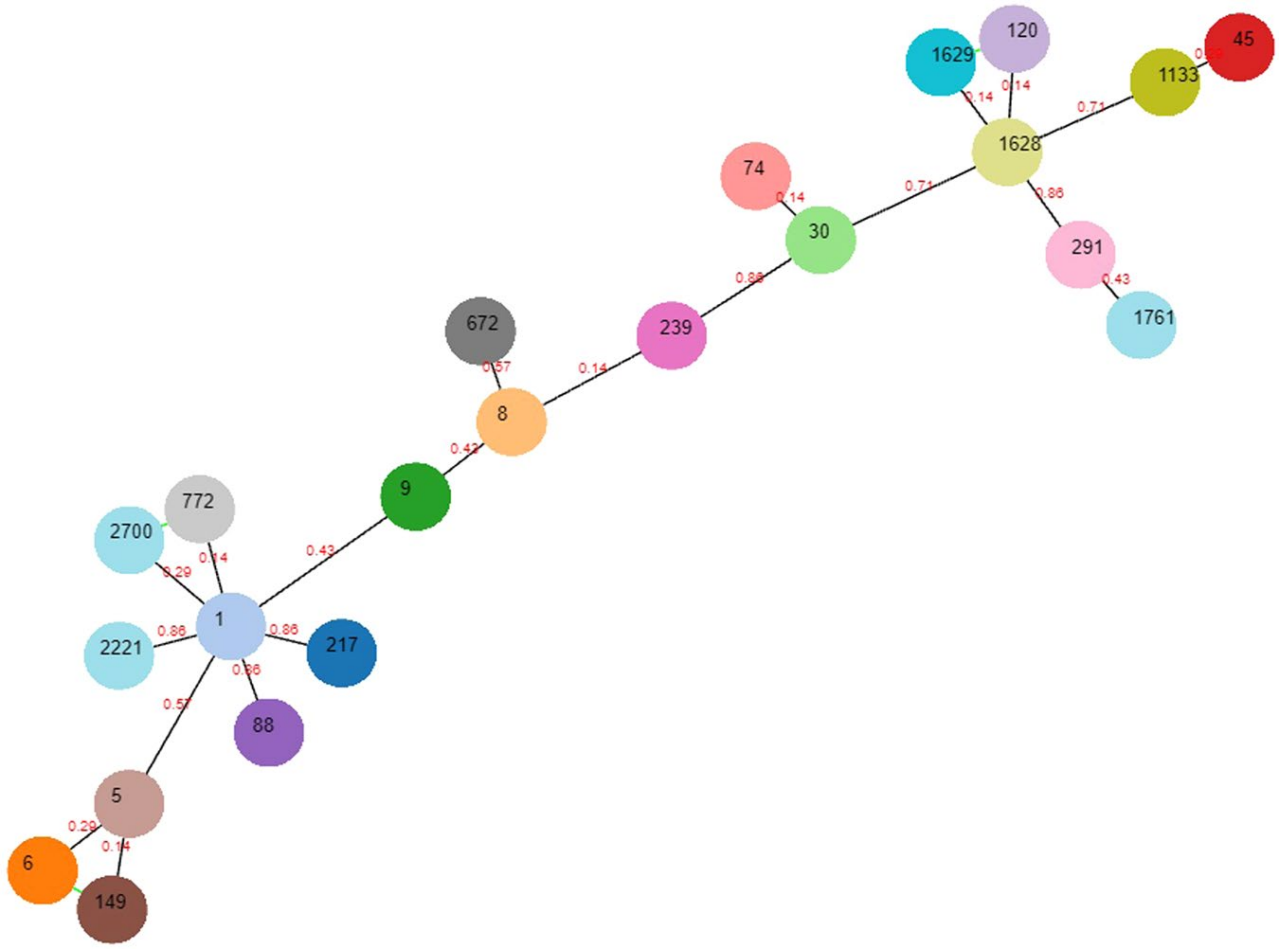
Phylogenetic relationship among 74 clinical isolates of *Staphylococcus aureus* using Sequence types (ST) obtained by Multi locus Sequence typing (MLST).

MLST analysis of the 74*S. aureus* clinical isolates determined 22 different sequence types (STs). ST1 (13.51%) was most common followed by ST672 (12.16%), ST217 (10.81%), ST120 (8.10%), ST30 (6.75%), ST239 (6.75%), ST5 (5.40%), ST8 (5.40%), ST1628 (5.40%), ST291 (4.05%), ST772 (4.05%), ST6 (2.70%) and ST9 (2.70%). Two major groups were identified using the goeBURST analysis (Fig. 1). The first and major group was around ST1 which was surrounded by 8 different STs while the second cluster was around ST1628. Rest of the STs were in singletons or doubletons indicating significant differences between these strains.

### *Agr* type I and III were predominant

*Agr* typing of the 74 clinical isolates showed type I (n = 32) and III (n = 18) to be the major types, followed by types IV (n = 12) and II (n = 10). One isolate displayed mixed type (I + II), and one was non-typeable.

### Clinical isolates of *S. aureus* exhibited strong, moderate and weak biofilm forming capacity

Biofilm forming capacity of the 74 clinical isolates was determined using CV assay. The majority (55%) of the isolates exhibited moderate biofilm forming capacity followed by 39% isolates which manifested strong biofilm production. Only 6% of the isolates formed weak biofilms. All the isolates formed black colonies on congo red agar plates.

### Characterisation of clinical isolates of MRSA

Antibiotic susceptibility assays against oxacillin and cefoxitin determined 15 isolates as methicillin-resistant (Table 2). Twelve MRSA clinical isolates were multi-drug resistant, and the remaining 3 were resistant to one or two classes of antibiotics. In 2 isolates, the *mecA* gene was not amplified, but they exhibited oxacillin resistance (MIC >4 mg/L). Therefore, the SCC*mec* typing of the 13 isolates revealed 7 as type III, followed by 3 isolates as type V, 2 isolates as IVc and 1 isolate was non-typeable (Table S1). Molecular typing of MRSA isolates revealed ST239 (33%) as the major sequence type followed by ST217 (26.66%). ST772 (13.33%), ST1 (6.66%), ST8 (6.66%), ST9 (6.66%), ST5 (6.66%) represent smaller proportions. Agr typing of these strains showed 66.67% isolates belonged to type I followed by 26.67% of type II and 6.67% of type III.

Majority of the MRSA isolates (60%) formed strong biofilm, followed by 33% moderate and only 6.6% of the isolates formed weak biofilm as determined by CV assay.

### Prevalence of Oxacillin susceptible *mecA* positive (OS-MRSA) isolates and role of *fem XAB* gene family

**Figure 2:**
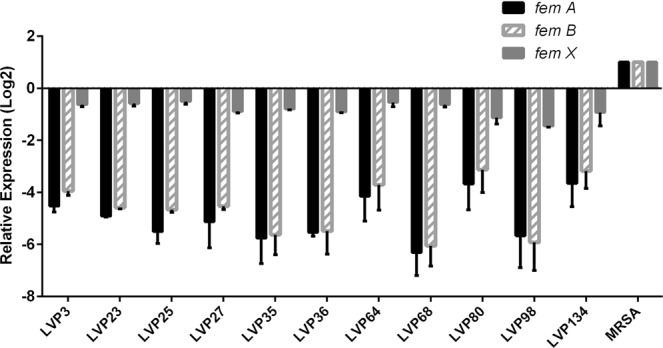
Relative gene expression analysis of the *fem XAB* family of genes in 11 OS-MRSA isolates using real-time PCR. The bar graph represents relative gene expression (Log_2_ ± SD).

Eleven *S. aureus* clinical isolates which were susceptible to oxacillin and cefoxitin and also positive for *mecA* gene and PBP2a agglutination were characterized as OS-MRSA (Table S1). Eighty one percent of the isolates were found resistant to one or two classes of antibiotic, and 18.2% were pan-sensitive. Sequence analysis of *fem XAB* genes in OS-MRSA isolates showed mutations in *fem* × (K3R, H11N, N18H and I51V) and *fem B* (L410F) gene, however, no mutations were found in *fem A*. Gene expression analysis revealed decreased expression of all the *fem* genes in comparison to MRSA (Fig. 2).

**Figure 3:**
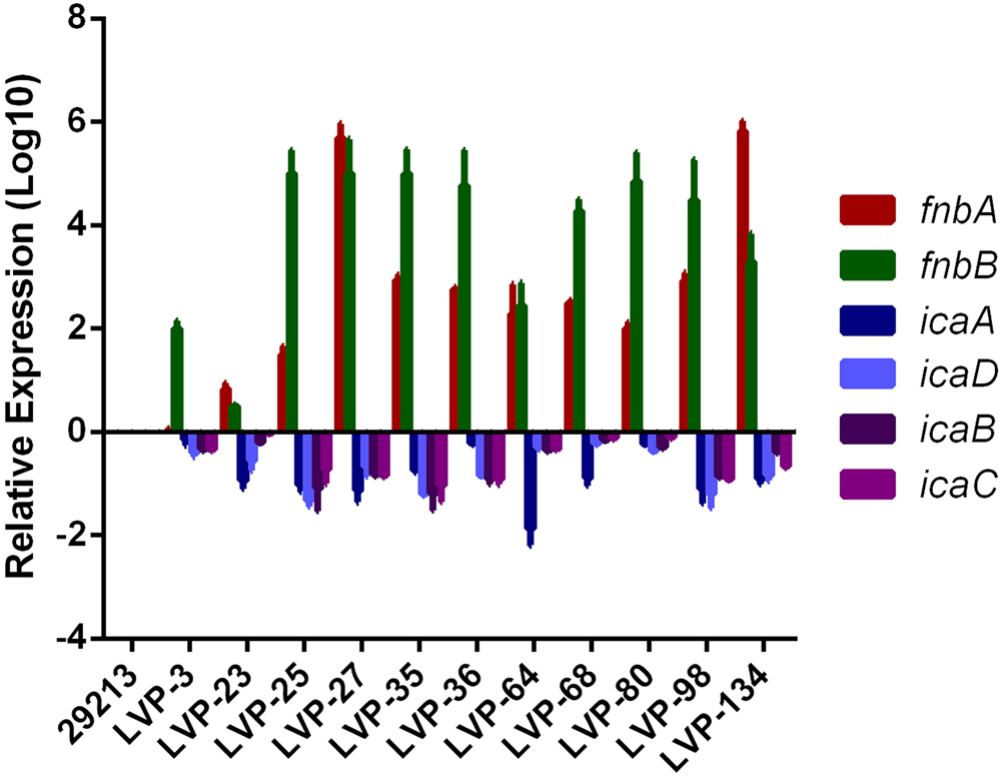
Relative gene expression analysis of biofilm pathway related genes*, icaADBC* and fibronectin binding proteins A & B in 11 OS-MRSA isolates. The bar graph represents gene expression levels (Log_10_ ± SD).

OS-MRSA isolates were genetically diverse and they belonged to 10 different STs represented by ST 30, ST120, ST772, ST1133, ST1, ST5, ST14, ST45, ST88, and ST217. SCC*mec* typing revealed Type V (n = 3), IVc (n = 2), IVa (n = 1), III (n = 2) and II (n = 2) in the group and one non-typeable. Major *agr* types appear to be III (54.5%) and I (18.2%), followed by type IV (18.2%) while one isolate was non-typeable (5%). CV assay revealed 45.45% to be moderate, and strong biofilm producers followed by 9.09% to be weak biofilm producer (Table S1). Gene expression analysis of *fnb* and *ica* genes involved in biofilm formation revealed up-regulation of *fnbA* and *B* genes in all OS-MRSA isolates whereas *icaABCD genes* were down-regulated (Fig. 3).

### Characterization of Methicillin Sensitive *S. aureus* (MSSA)

Isolates with oxacillin MIC of ≤2 mg/L were categorized as methicillin-sensitive *S. aureus* (MSSA, n = 48) (Table 2). MSSA isolates were genetically diverse with 17 different STs type represented by ST672, ST1, ST120, ST1628, ST8, ST291, ST217, ST5, ST6, ST30, ST1629, ST1761, ST2700, ST2221, ST74, ST9 and ST149.

Majority of the isolates belonged to a*gr* type I (39.6%) and III (25%) followed by types IV (20.8%), II (10.4%), and type I & II (2.08%) (Table S1). Predominantly MSSA isolates showed moderate biofilm (64.6%) formation followed by strong (31.25%) and weak (4.17%) biofilm phenotype.

## Discussion

The present study represents the molecular variability and antibiotic susceptibility profiles of *S. aureus* clinical isolates causing ocular infections. Antibiotic susceptibility determined majority of the isolates as resistant to one or two classes of antibiotics, followed by MDR and pan sensitive clinical isolates. These clinical isolates were further characterized as methicillin-sensitive or resistant based on the phenotypic and genotypic tests. MRSA in ocular infections had been previously reported from countries such as Taiwan (57.6%), China (52.8%), India (43%), United States (41.6%) and Brazil (9.9%)^2,23–26^. We found 17.6% MRSA (n = 15) in our study and two MRSA without the *mecA* gene, which is in line with previous findings where 9.8% of MRSA isolates were *mecA* negative^27^.

The present finding also highlighted a 14.86% prevalence rate of OS-MRSA clinical isolates which were not reported earlier from ocular infections in India. OS-MRSA has surfaced across the globe and reported from human and animal infections^2,3^. We also found 9 oxacillin susceptible clinical isolates which did not produce PBP2a but were *mecA* PCR positive^28^. OS-MRSA can be misinterpreted if only either phenotypic or genotypic test is used and may have serious implications^29^. Earlier reports of OS-MRSA have shown mutations in the *fem XAB* genes^20,30^. Consistent with the previous finding by Giannouli *et al*., 2010, we found mutations in the *femX* (K3R, H11N, and N18H) and the *femB* (L410F) gene. An additional new mutation in the *femX* gene (I51V) was identified. The downregulation of *fem XAB* genes was seen, in contrast to earlier observation where no change has been reported^20^. Methicillin resistance is caused by changes associated with genes involved in cell wall metabolism and the *fem* gene family are also involved in cell wall biosynthesis, hence, decreased expression of the *fem* genes may lead to oxacillin sensitivity in case of OS-MRSA isolates^31^. Increased expression of the *femA* gene has been reported in high-level MRSA isolates which were comparatively found to be reduced in low-level MRSA followed by MSSA isolates^32^. Therefore, our study indicates the decreased expression of the *fem* genes, may be responsible for hypersusceptibility towards oxacillin in OS-MRSA isolate^20^. However, the underlying mechanism operative in OS-MRSA is still obscure and need investigation.

Further, molecular typing of the clinical isolates showed diversity among them. We found ST239 is a predominant lineage in MRSA isolates followed by ST217 and ST772^33–35^. The ST239 clinical isolates were mostly reported in hospital-associated infections from India and other Asian countries, while ST217 and ST772 were reported from community-acquired MRSA infections^36,37^. ST772 is mainly reported from the southern part of India and is being considered to be replacing ST239 in hospitals^37^. Recently, ST217 has been identified as a new emerging clone in both hospital and community-acquired infections from India^37^. Along with their resistant phenotype, the add-on capacity to form biofilms assist their prolonged survival by enhanced tolerance against host mechanisms or antibiotics^14,38^. Therefore, we also studied the biofilm forming capacity of the clinical isolates with variable methicillin phenotype in an attempt to establish any link with strain lineages and phenotype.

The majority of the MRSA formed strong biofilm (n = 9), and predominantly belonged to ST239 (n = 5) with SCC*mec* type III (n = 4) and V (n = 1). ST239 MRSA causing ocular infections have been previously reported to be from Taiwan (29.4%), although its biofilm phenotype was not evaluated^26^. Strong biofilm forming ST239 isolates were also reported from other countries like Korea, Brazil, and India^39–41^. We also came across ST772 MRSA with SCC*mec* type V, (n = 2) which formed a strong biofilm and has been considered as an emerging threat responsible for disease outbreaks^42^. One of the *mecA* negative MRSA belonging to ST5, also formed a strong biofilm. The present findings suggest that MRSA isolates with SCC*mec* types III and V may be dominant biofilm producers. A recent study shows that SCC*mec* types III & V display higher adhesion and are less hydrophilic in comparison to SCC*mec* type II^40^. However, there can be a difference in the observed phenomenon when compared *in vivo*. Biofilm formation have been known to be dependent on *icaADBC* operon in MSSA isolate, whereas in MRSA, *fnb* genes are involved^43^. The biofilm mechanism in OS-MRSA isolates has not been studied earlier. Our data reveal that biofilm formation in OS-MRSA isolates is *icaADBC* independent and regulated via *fnb* genes.

*Agr* is studied mostly as virulence locus but recently shown to be involved in biofilm formation^44^. Genotyping and transcriptional analysis to establish a direct correlation between biofilm formation and *agr* have been done in recent times^44^. In contradiction to the expected decreased biofilm forming capacity of isolates with *agr* type I, we found isolates (5 MRSA; 2MSSA) with *agr* type I forming strong biofilms^44^. Therefore, it would be interesting to study such isolates and understand their mechanism of biofilm production. Isolates with type III *agr* are known to induce moderate biofilms, however, our type III *agr* isolates showed strong biofilm phenotype^44^. Similar to previous findings, MRSA isolates belonging to ST772/SCC*mec* type V and *agr* type II and a *mecA* negative, ST5 isolates produced strong biofilms^44^. Our findings interestingly support the recent reports where methicillin resistance effects *icaABCD* and *agr* phenotype thereby altering expected biofilm phenotype^43,45^. Also 12 out of 15 MRSA isolates were MDR out of which 8 were forming strong biofilms. A recent publication also stated that 87% of hospital-acquired ocular MRSA infection were MDR^46^. This is an alarming situation because treating a MDR bacteria forming strong biofilm makes the treatment extremely difficult.

OS-MRSA isolates with strong biofilm (n = 5) belonged to variable ST types, but all belonged to *agr* type III. There seems to be hardly any study on the biofilm phenotype of the newly emerging class of OS-MRSA isolates, and hence this could shed some light on our understanding of these isolates. OS-MRSA isolates which could be misinterpreted as sensitive isolates with an add-on ability to form strong biofilm may pose a dangerous threat to public health. Another fact to note was that MSSA isolates belonging to *agr* IV and III appeared to form a strong biofilm.

Our data suggest no association of biofilm intensity with their antibiotic susceptibility phenotype. However, the variable biofilm forming ability might be linked with the differences in their metabolic and biosynthetic processes as evident in the case of *Enterococcus faecalis* isolates^47^. Hence, it will be interesting to do comparative studies exploring the biofilm mechanism among strong, moderate and weak biofilm isolates which may help us in identifying drug targets against biofilm-forming bacteria. However, any resistant strain with strong biofilm forming capacity will be an immense threat to patients with ocular infections and in other diseases too. Further, with the increasing prevalence of OS-MRSA strains in various infections across the globe, studies are warranted to understand this peculiar phenotype.

## Supplementary information

Table S1
