# Supplementary material for: Decreased expression of femXAB genes and fnbp mediated biofilm pathways in OS-MRSA clinical isolates
Source: Sci Rep. 2019 Nov 5;9:16028. doi: 10.1038/s41598-019-52557-z (PMC6831631; doi:10.1038/s41598-019-52557-z)
Supplement: Supplementary file 1 — Table S1 [file 41598_2019_52557_MOESM1_ESM.docx]

**Decreased expression of *femXAB* genes and *fnbp* mediated biofilm pathway in OS-MRSA clinical isolates**

Umarani Brahma^1^, Paresh Sharma^1^, Shweta Murthy^1^, Savitri Sharma^2,^ Shalini Chakraborty^1^, Sundarapu Naga Appalaraju^1^, Vasundhra Bhandari^1^

**Supplementary Table S1: Characterization of 74 clinical isolates of *Staphylococcus aureus***

| Isolate ID | Disease | Clinical Specimen | Year | Methicillin Phenotype | Antibiotic profile | Sequence Type (ST)/*agr* type | Biofilm Type |
| --- | --- | --- | --- | --- | --- | --- | --- |
| 1. LVP-2 | Microbial keratitis | Corneal scraping | 2015 | MRSA | MDR | ST239/I | Strong |
| 1. LVP-7 | Microbial keratitis | Corneal scraping | 2015 | MRSA | MDR | ST239/I | Strong |
| 1. LVP-8 | Lid electric burn | Pus | 2015 | MRSA | Res | ST239/I | Strong |
| 1. LVP-11 | Preseptal cellulitis | Pus | 2015 | MRSA | Res | ST217/I | Moderate |
| 1. LVP-12 | Basal cell carcinoma | Pus | 2015 | MRSA | Res | ST217/I | Moderate |
| 1. LVP-17 | Preseptal cellulitis | Pus | 2015 | MRSA | MDR | ST217/I | Moderate |
| 1. LVP-18 | Orbital cellulitis | Pus | 2015 | MRSA | MDR | ST239/I | Strong |
| 1. LVP-42 | Microbial keratitis | Corneal scraping | 2015 | MRSA | MDR | ST239/I | Strong |
| 1. LVP-66 | Lid abscess | Pus | 2016 | MRSA | MDR | ST772/II | Strong |
| 1. LVP-69 | Microbial keratitis | Corneal scraping | 2016 | MRSA | MDR | ST772/II | Strong |
| 1. LVP-77 | Conjunctivitis | Conjunctival swab | 2016 | MRSA | MDR | ST1/III | Strong |
| 1. LVP-85 | Conjunctivitis | Conjunctival swab | 2016 | MRSA | MDR | ST8/I | Moderate |
| 1. LVP-89 | Microbial keratitis | Corneal scraping | 2016 | MRSA | MDR | ST9/II | Weak |
| 1. LVP-132 | Microbial keratitis | Corneal scraping | 2016 | MRSA | MDR | ST217/I | Moderate |
| 1. LVP-135 | Microbial keratitis | Corneal scraping | 2016 | MRSA | MDR | ST5/II | Strong |
| 1. LVP-3 | Microbial keratitis | Corneal scraping | 2015 | OS-MRSA | Sen | ST1133/I | Moderate |
| 1. LVP-23 | Conjunctivitis | Conjunctival swab | 2015 | OS-MRSA | Res | ST14/III | Strong |
| 1. LVP-25 | Microbial keratitis | Corneal scraping | 2015 | OS-MRSA | Res | ST5/III | Strong |
| 1. LVP-27 | Buckle surgery | Pus | 2015 | OS-MRSA | Sen | ST88/III | Strong |
| 1. LVP-35 | Microbial keratitis | Corneal scraping | 2015 | OS-MRSA | Res | ST1/III | Moderate |
| 1. LVP-36 | Lid abscess | Pus | 2015 | OS-MRSA | Res | ST772/NT | Moderate |
| 1. LVP-64 | Microbial keratitis | Bandage contact lens | 2016 | OS-MRSA | Res | ST30/III | Strong |
| 1. LVP-68 | Injury | Foreign body | 2016 | OS-MRSA | Res | ST45/IV | Weak |
| 1. LVP-80 | Orbital cellulitis | Pus | 2016 | OS-MRSA | Res | ST120/IV | Moderate |
| 1. LVP-98 | Microbial keratitis | Corneal scraping | 2016 | OS-MRSA | Res | ST217/I | Moderate |
| 1. LVP-134 | Microbial keratitis | Corneal scraping | 2016 | OS-MRSA | Res | ST30/III | Strong |
| 1. LVP-1 | Microbial keratitis | Corneal scraping | 2015 | MSSA | Res | ST672/I | Weak |
| 1. LVP-4 | Microbial keratitis | Corneal scraping | 2015 | MSSA | Res | ST1629/IV | Strong |
| 1. LVP-5 | Post-surgery infection | Pus from anterior chamber | 2015 | MSSA | Res | ST149/II | Moderate |
| 1. LVP-6 | Stitch granuloma after tarsal frontal sling surgery | Conjunctival swab | 2015 | MSSA | Res | ST-672/I | Moderate |
| 1. LVP-9 | Lid reconstruction surgery wound infection | Pus | 2015 | MSSA | Res | ST1/III | Moderate |
| 1. LVP-10 | Microbial keratitis | Corneal scraping | 2015 | MSSA | Res | ST120/II | Moderate |
| 1. LVP-13 | Lacrimal abscess | Pus | 2015 | MSSA | Res | ST672/I | Moderate |
| 1. LVP-15 | Pyogenic granuloma | Pus | 2015 | MSSA | Res | ST672/I | Moderate |
| 1. LVP-16 | Microbial keratitis | Corneal scraping | 2015 | MSSA | Sen | ST672/I | Strong |
| 1. LVP-19 | Microbial keratitis | Corneal scraping | 2015 | MSSA | Res | ST672/1 | Moderate |
| 1. LVP-20 | Orbital abscess | Pus | 2015 | MSSA | Res | ST1628/IV | Strong |
| 1. LVP-22 | Infected socket | Pus | 2015 | MSSA | Sen | ST291/I | Moderate |
| 1. LVP-24 | Preseptal abscess | Pus | 2015 | MSSA | Res | ST74/III | Strong |
| 1. LVP-26 | Orbital cellulitis | Pus | 2015 | MSSA | Res | ST217/I | Strong |
| 1. LVP-29 | Microbial keratitis | Corneal scraping | 2015 | MSSA | Res | ST120/IV | Moderate |
| 1. LVP-30 | Post-tarsorrhaphy infection | Eye discharge | 2015 | MSSA | Res | ST1628/IV | Moderate |
| 1. LVP-32 | Microbial keratitis | Corneal scraping | 2015 | MSSA | Res | ST1/III | Moderate |
| 1. LVP-33 | Conjunctivitis | Conjunctival swab | 2015 | MSSA | Res | ST1/III | Moderate |
| 1. LVP-37 | Lacrimal abscess | Pus | 2015 | MSSA | Sen | ST8/I | Moderate |
| 1. LVP-38 | Orbital mass with sinus | Pus | 2015 | MSSA | Res | ST1/III | Moderate |
| 1. LVP-60 | Microbial keratitis | Corneal scraping | 2016 | MSSA | Res | ST672/I | Moderate |
| 1. LVP-61 | Panophthalmitis | Pus | 2016 | MSSA | Res | ST672/I | Moderate |
| 1. LVP-72 | Microbial keratitis | Corneal scraping | 2016 | MSSA | Res | ST291/I | Moderate |
| 1. LVP-78 | Microbial keratitis | Corneal scraping | 2016 | MSSA | Res | ST672/I | Moderate |
| 1. LVP-86 | Endophthalmitis | Vitreous | 2016 | MSSA | Res | ST1/III | Moderate |
| 1. LVP-87 | Microbial keratitis | Corneal scraping | 2016 | MSSA | Res | ST2221/III | Strong |
| 1. LVP-88 | Lacrimal abscess | Pus | 2016 | MSSA | Res | ST1628/I | Weak |
| 1. LVP-90 | Panophthalmitis | Pus | 2016 | MSSA | Res | ST1/III | Moderate |
| 1. LVP-91 | Upper brow abscess | Pus | 2016 | MSSA | Res | ST30/III | Moderate |
| 1. LVP-93 | Panophthalmitis | Pus | 2016 | MSSA | Res | ST291/I | Moderate |
| 1. LVP-94 | Microbial keratitis | Corneal scraping | 2016 | MSSA | Res | ST9/II | Moderate |
| 1. LVP-96 | Microbial keratitis | Corneal scraping | 2016 | MSSA | Sen | ST217/I | Moderate |
| 1. LVP-97 | Post-surgery infection | Pus | 2016 | MSSA | Res | ST1/III | Moderate |
| 1. LVP-99 | Endophthalmitis | Vitreous | 2016 | MSSA | Res | ST217/I | Moderate |
| 1. LVP-101 | Open globe injury | Iris and exudative membrane | 2016 | MSSA | Res | ST1628/IV | Strong |
| 1. LVP-103 | External hordeolum | Pus | 2016 | MSSA | Res | ST30/III | Strong |
| 1. LVP-107 | Lacrimal abscess | Pus | 2016 | MSSA | Res | ST8/I | Moderate |
| 1. LVP-108 | Post-surgery tunnel infection | Tunnel scraping | 2016 | MSSA | Res | ST8/I+II | Strong |
| 1. LVP-109 | Socket infection | Pus | 2016 | MSSA | Sen | ST6/I | Moderate |
| 1. LVP-110 | Panophthalmitis | Pus | 2016 | MSSA | Res | ST5/II | Strong |
| 1. LVP-114 | Microbial keratitis | Corneal scraping | 2016 | MSSA | Res | ST5/II | Strong |
| 1. LVP-115 | Microbial keratitis | Corneal scraping | 2016 | MSSA | Res | ST2700/I | Moderate |
| 1. LVP-116 | Preseptal cellulitis | Pus | 2016 | MSSA | Res | ST120/IV | Strong |
| 1. LVP-118 | Microbial keratitis | Corneal scraping | 2016 | MSSA | Res | ST120/IV | Strong |
| 1. LVP-120 | Microbial keratitis | Corneal scraping | 2016 | MSSA | Res | ST6/I | Moderate |
| 1. LVP-124 | Orbital tumor | Biopsy | 2016 | MSSA | Res | ST1761/IV | Moderate |
| 1. LVP-129 | Microbial keratitis | Corneal scraping | 2016 | MSSA | Res | ST120/IV | Strong |
| 1. LVP-130 | Microbial keratitis | Corneal scraping | 2016 | MSSA | Res | ST1/III | Strong |

MDR refers to isolates resistant against three or more class of antibiotics

Res refers to resistance to one or two class of antibiotics

Sen refers to susceptible to all antibiotics

* MRSA denotes: Methicillin Resistant *S. aureus*

OS-MRSA denotes: Oxacillin Susceptible *mecA* positive *S. aureus,*

MSSA denotes: Methicillin Sensitive *S. aureus,*

NT denotes: Non-typeable
